# Understanding how young people become motivated to take their human immunodeficiency virus medication (antiretroviral therapy) and how the need for adherence is communicated

**DOI:** 10.4102/hsag.v25i0.1458

**Published:** 2020-12-14

**Authors:** Warren Hickson, Pat M. Mayers

**Affiliations:** 1 Department of Public Health and Family Medicine, Faculty of Health Sciences, University of Cape Town, Cape Town, South Africa; 2 Department of Health and Rehabilitation Sciences, Faculty of Health Sciences, University of Cape Town, Cape Town, South Africa

**Keywords:** adolescents, young people, ART adherence, health communication, HIV, South Africa

## Abstract

**Background:**

Antiretroviral therapy (ART), the only effective treatment for human immunodeficiency virus (HIV), requires excellent long-term compliance. Poor levels of adherence to ART, especially amongst adolescents and young adults in South Africa, have been reported.

**Aim:**

This study aimed to explore how young people become motivated to take their HIV medication (ART) and how the need for adherence is communicated.

**Setting:**

The study was conducted in a peri-urban township in the Western Cape, South Africa.

**Methods:**

A qualitative grounded theory approach was employed. Eighty young people were purposively recruited. Participant observation, focus groups and semi-structured interviews were utilised to explore how effective ART adherence messages are in motivating adherence amongst young people and how they would like ART adherence to be communicated to them. All interviews and focus groups were transcribed and analysed by using cross-comparison analysis. Measures to ensure trustworthiness were established and ethical considerations were adhered to.

**Results:**

Young people’s adherence motivation was an outcome of reconnecting to one or more trusted significant other(s) from within their belonging group, who accepted and supported them, which in turn affirmed their prior belonging identities of son, daughter, other family member or close friend. This facilitated reconnection to their present and future hopes, which in turn increased their motivation to live and to adhere to treatment.

**Conclusion:**

The findings highlight the need for the development of more effective communication strategies, which facilitate and support young people’s reconnection to trusted members of their belonging groups, and also help belonging group members to accept, affirm and support adherence.

## Introduction and background

South Africa has the largest human immunodeficiency virus (HIV) epidemic in the world, with an estimated 7.2 million people living with HIV (PLWH) (UNAIDS 2018). Young people are particularly vulnerable to HIV infection and account for approximately 45% of all new HIV infections worldwide (UNAIDS 2018). Antiretroviral therapy (ART), the only effective treatment for HIV, requires strict patient adherence. Poor levels of ART adherence amongst adolescents and young adults in South Africa have been reported (Hornschuh et al. 2017; Zanoni et al. 2016), with a 50% increase in HIV-related deaths in this group from 2005 to 2012 (Lall et al. 2015).

Effective communication and the need for youth-centred interventions to promote long-term optimal ART adherence have been prioritised in the South African National Strategic Plan on HIV, tuberculosis (TB) and sexually transmitted infections (STIs) during 2017–2022 (National Department of Health 2017). A number of health professionals, including doctors, nurses and pharmacists in the ART clinics, communicate ART adherence messages to young people; however, trained lay counsellors play a pivotal role in HIV testing and in initiating young people onto ART and are the main channel of communication for adherence promotion (Gouse et al. 2018).

Health communication behavioural change interventions are informed by health behaviour models that emphasise that human behaviour is influenced by the beliefs, expectations, attitudes, motives and perceptions attributed to a specific behavioural action (Glanz, Rimer & Viswanath 2008). These models focus on the process of thinking, reasoning, hypothesising and expecting and seek to shift a person’s thinking so that behaviours to avoid, prevent or limit the disease are valued (Champion & Skinner 2008). The main models used to inform HIV interventions and ART communication throughout sub-Saharan Africa are the Health Belief Model (HBM) (Janz & Becker 1984), the Theory of Reasoned Action (TRA) (Terry, Gallois & McCamish 1993), the Theory of Planned Behaviour (TPB) (Ajzen 1991), the Extended Parallel Process Model (EPPM) (Witte 1992) and Social Cognitive Theory (SCT) (Bandura 1998).

These models are based on the hypothesis that an individual’s health behaviour is determined by the value that he or she places on a health-related goal and the evaluation of whether a particular behavioural action will achieve that goal (Glanz et al. 2008). The models have been useful in identifying factors that influence health behaviour, such as cognitive processes, attitudes, fear, beliefs, perceptions of vulnerability and benefits (Dutta-Bergman 2005). Although these models have often been used to underpin the design and implementation of HIV prevention campaigns, these have not been widely adopted for communication interventions designed to inform and motivate adherence to ART and have had a limited impact (Munro et al. 2007).

The HBM has limited applicability to explain the complexity of sexual behaviour, and interventions informed by this model are unlikely to achieve significant behavioural change (Hounton, Carabin & Henderson 2005). Framing perceived susceptibility and severity as predictors of health behaviour may not necessarily apply to every person’s experience of a particular health threat; this results in sections of the intended audience disregarding the communicated health messages (Zak-Place & Stern 2004). Munro et al. (2007) argue that the HBM, EPPM and TRA models are too simplistic, framing adherent behaviour as a series of rational decisions that need to be incorporated into a person’s daily schedule. The complexity of what it means to live with HIV, in the context of localised cultural beliefs and stigma that can significantly influence a person’s adherence motivation, is underestimated (Mutwa et al. 2013).

The Information–Motivation–Behavioural skills (IMB) model incorporates the broader complexities of human behaviour into the context of long-term adherence (Fisher, Fisher & Harman 2003). Building on the principles of SCT, the IMB model proposes that information, motivation and behavioural skills are the main determinants of self-efficacy. Information should clearly explain the medicine regime, including regular medication times and the potential medicine side effects. Motivation also includes attitudes and beliefs about the potential outcomes of optimal and suboptimal adherence and patients’ perceptions of how significant others could support them to achieve optimal adherence. For a successful ART adherence, behavioural skills training is also needed, for example medication reminders, incorporating the ART regime into daily routines, strategies to minimise side effects and understanding how to acquire social support (Peltzer et al. 2010). Studies using this model have been conducted with PLWH (Norton et al. 2010; Zeleke 2015) with varying results. Amico et al. (2018) argue that medication adherence is determined by a set of complex factors, which can be addressed by using social behavioural models but highlight the need to tailor broad model constructs to the level of the intervention target.

It was within this context that this study aimed to explore how young people become motivated to adhere to ART and how this should be communicated to them.

## Study setting

Khayelitsha, Cape Town’s largest township, is located approximately 35 km from Cape Town city centre. Khayelitsha has the highest HIV prevalence in the Western Cape Province, with 34.3% of pregnant women being HIV-positive in 2012 compared with 29.5% nationally (Stinson et al. 2017). Five healthcare facilities in the Khayelitsha health district, which provide adolescent and youth HIV services, were selected as study sites: the district hospital, three primary care centres (identified as Clinics 1, 2 and 3) and an international health non-governmental organisation (NGO) that was supporting young people in the area who were living with HIV.

## Methods

### Research design

A qualitative grounded theory approach (Charmaz 2014) was utilised for the purpose of this study, to conduct a series of focus groups, semi-structured interviews and observations with young people aged 16–24 to explore how they become motivated to ART adherence and how they think adherence should be communicated.

### Sampling and data collection

#### Focus groups and interviews

Participants for the focus groups were purposively sampled, by using a mediated access approach, at each of the study sites. Four focus group (FG) interviews were conducted (*n* = 80) in a private room in either the NGO office or the clinic. Ten semi-structured interviews were conducted in settings, which suited the participants and offered privacy with minimal noise background (Table 1). For the focus groups, English was used to frame the questions, and the discussion was in both English and isiXhosa. A Xhosa-speaking research assistant translated the isiXhosa discussion.

**TABLE 1:** Participants in focus groups and follow-up interviews.

	Participants	Characteristics of participants
Focus group 1: NGO office	Young men (*n* = 5)	16- to 22-year-old males and females who were living with HIV (prenatally or behaviourally infected). All were prescribed ART and were regular attendees of the support group at Clinic 2.
Focus group 2: NGO office	Young women (*n* = 8)	
Interviews following focus group: NGO office	Young people (*n* = 2)	22-year-old man, prenatally infected with HIV, had been taking ART for several years.
		21-year-old woman, behaviourally infected with HIV, recently prescribed ART.
Focus group 3: Clinic 2	Young people (*n* = 39)	16- to 24-year-old persons living with HIV: 38 women and one young man
Focus group 4: Clinic 2	Young people (*n* = 20)	16- to 24-year-old persons of mixed status: 14 men and 6 women
Semi-structured interviews	Young men (*n* = 8)	Young men attending Clinic 3 for an HIV test

ART, antiretroviral therapy; NGO, non-governmental organisation; HIV, human immunodeficiency virus.

Each focus group began with the opening question: ‘These are the messages that clinicians and healthcare workers use to explain ART adherence, do they help motivate you to take your treatment?’ (Table 2).

**TABLE 2:** Antiretroviral therapy message components and content captured during participant observation.

Message component	Message content
ART offers hope	The benefit of treatment in response to the severity of HIV infection – you can live a normal life.
ART medication adherence	Choosing a time(s) to take your pills – You must take them at the same time every day.
Disclose for adherence support	Find a treatment buddy, someone who can support your adherence.
Living a healthy lifestyle	You must continue to wear a condom, eat healthily and live with minimal stress.
What you must do to access linkage to care?	How to access treatment, an initial CD4 cell count test and on-going care.
Join an adherence club	You may be able to join an adherence club.

ART, antiretroviral therapy; HIV, human immunodeficiency virus.

Theoretical sampling was used to invite focus group participants, who had demonstrated a clear articulation of the issues discussed in the focus groups, to participate in an individual follow-up interview. Interviews of approximately 30 min were conducted in English, audio-recorded and transcribed.

#### Participant observation

In this phase, the first researcher spent time in each of the three HIV clinics to observe how adherence messages were communicated by healthcare workers at the different stages of HIV healthcare: the initial wait for the HIV test in the clinic, the HIV test itself, communicating the test results, ART initiation-counselling sessions and pill dispensing (Table 3). On three occasions, the first researcher was tested and counselled, which proved helpful in generating open conversation amongst participants. Field notes were kept and incorporated into the data analysis.

**TABLE 3:** Participant observation of the different stages of HIV healthcare engagement.

Stage of healthcare engagement	Healthcare worker	Description of event(s)
Testing, receiving a diagnosis and post-test counselling	HIV counsellor, Clinic 3 and hospital	The first researcher attended Clinic 3 for an HIV test. The researcher followed the testing process: waited his turn with other men, was tested, waited for the result, obtained the test result and was counselled by the nurse.
ART initiation sessions (C1, C2, C3)	HIV counsellor, Clinic 1	The first researcher attended, with young people, three ART initiation-counselling sessions.
HIV clinic waiting room	Clinic 3	The first researcher sat in the clinic waiting room listening and was involved in conversations about being tested and waiting for test results.

ART, antiretroviral therapy; HIV, human immunodeficiency virus.

#### Data analysis

Grounded theory commences with the process of collecting rich data from research participants (Charmaz 2014). A first layer of data was gathered and analysed, following which cross-comparison across the data by using a coding system commenced. Line-by-line coding was used to name words, lines or segments of data, followed by focussed coding. Significant initial codes were selected, and participants’ experiences and stories were compared across data sources to separate, sort, synthesise and compare data. Codes with similar properties were merged into larger categories (Charmaz 2014). Continual cross-comparison of data facilitated clarification of meanings. Reflective notes and theoretical memos were utilised, which enabled the next phase of theoretical sampling (Charmaz 2014). Emerging categories were reviewed for new patterns of experience within the narrative, and data saturation was determined after no new patterns emerged.

#### Trustworthiness

The quality, credibility and transferability of the study were ensured through a prolonged engagement with participants, detailed audit trail, multiple sources of data and comparison of themes (Kornbluh 2015).

#### Ethical considerations

Ethics approval for the study was granted by the University of Cape Town Faculty of Health Sciences Human Research Ethics Committee (Ref: HREC 061/2011) and renewed over the period of the study. To include young people below the statutory age of consent (18 years), special ethics approval was obtained to interview 16- to 18-year-old participants with unassisted consent. The ethical considerations of voluntary participation, informed consent, confidentiality and the right to autonomy, justice and minimisation of risk were adhered to.

## Findings

Three stages of young people’s journeys to become motivated to adhere to ART emerged: the response to a positive HIV test result (diagnosis of trauma); coming to terms with being HIV-positive (acceptance and support); and learning about ART and adhering to it (learning to live with HIV).

### Trauma upon diagnosis of human immunodeficiency virus

The trauma of a person diagnosed as HIV positive engendered a fear of illness, early death, rejection by a partner, family and friends and a loss of hope. Healthcare workers encouraged participants that there was hope as HIV is a manageable and treatable chronic illness. Despite this encouragement, the young people were unable to absorb the messages, as the shock at the positive HIV results made it almost impossible to ‘hear’ and comprehend the information:

‘When you first receive the HIV-positive results, first of all you don’t listen – whatever comes out after you were told that you’re HIV-positive, you don’t listen …’ (Focus group [FG] 2, Participant [P] 21, young woman living with HIV [YWLWH], age 19)

For one young woman, however, her diagnosis of being HIV positive brought a sense of relief and comfort. Perinatally infected, she had lost her mother to an acquired immune deficiency syndrome (AIDS)-related illness:

‘Ja … I was happy, because my mother died when I was young, and I grew up with my grandmother. I’ve always been sick, so when I know that I was HIV-positive, I was happy, because everyone didn’t find the reason why I got sick every time I didn’t go to school, because I was sick. So, I was happy, because I could find the reason why I’m always sick.’ (FG 2, P16, YWLWH, age 22)

Three components of trauma upon diagnosis of HIV experienced by participants emerged.

#### The fear of rejection and loss of identity

The trauma that young people suffered upon receiving their diagnosis of HIV was linked to a fear of rejection by significant others, in particular the fear of no longer being worthy of belonging to a family and close peer group, which left them feeling hopeless. For this young man, awaiting his test result at the clinic, an HIV-positive diagnosis signified the end of his world:

‘I’d lose my girlfriend, and then no woman would ever want to be with me ever again, then I could not have children, and no one would ever give me a job and I will never be able to buy a house … my life would be over!.’ (Interview, Clinic 3, P 81, young man [YM], age 19)

This overwhelming fear left participants with a sense of immense loss that life was over and that family and friends would reject them.

#### The shock of being infected by someone

The shock of being told they had been infected was compounded by the realisation that they had been infected by a trusted other: ‘Many of us are shocked because we trust our partners and think, why did they not tell me!’ (Interview, Clinic 3, P82, M, age 20).

Participants felt enormous betrayal that a trusted sexual partner, or someone they had with whom they had been intimate, had infected them and anger that a partner had chosen not to disclose his or her status whilst continuing to engage in unprotected sex.

#### The fear of becoming sick and dying

An HIV-positive diagnosis generated fear of sickness and early death. Despite the reassurance of effective treatment from their healthcare providers, the diagnosis re-activated memories of parents, siblings, friends and community members whom they had lost through HIV: ‘It’s like a death sentence, we get afraid of being sick and dying’ (FG 1, P3, young man living with HIV [YMLWH], age 23).

### Acceptance and support

In the focus groups, the question ‘What you would have wanted to hear at the time of initial diagnosis?’ was explored. A 16-year-old young man, with tears in his eyes, stated: ‘… it’s simple, the answer is love’. The participants’ most important need after diagnosis was to receive acceptance and support from family members. Disclosure of their newly diagnosed status to their families was for some participants very challenging, but others were reassured of their family’s love and acceptance. A YWLWH described how, immediately after her diagnosis, she had longed for the support of her mother, who had died of AIDS. With her cousin’s support, she had been able to inform her uncle about her test result who, despite her initial apprehension about his response, had also supported her. For some participants, however, their fear of rejection was associated with punishment:

‘… it’s sort of when, it’s when you are growing and sometimes you are naughty, and you are stealing some people’s stuff. That I have done something wrong, they be angry at home for me. They say no, go out, we don’t want you here, you’re just a cruel person who is just a criminal.’ (FG 6, P 78, YMLWH, age 21)

Group support was vital for participants, who chose to go to the clinics in groups to be tested so they could support one another, particularly if there was concern about the family response, as explained by young men in a focus group (FG 6):

‘I go with my friends, like go to the same room with them, they must know … like they must know my status. We always open to each other … to support each other. We talk about this every day.’ (Fg 6, age 19, Clinic 3)

‘Like, if they are my friends, there’s no need to leave them behind. You see I must wait for them …’ (P62, FG 6, age 20, Clinic 3)

‘Ja, my friend, I can’t leave them behind.’ (P57, FG 6, age 20, Clinic 3)

‘… like when you come to the clinic, I don’t care about the results …’ (P60) ‘You see the thing is, we came as a group, all five, you and me, and me positive and him and I negative … support that you came as friends and you go as friends.’ (P62, Clinic 3, age 20)

A participant who attended the clinic with a close friend was assured of support if the diagnosis was positive:

‘… you know … if you’re going the first time. When you go home … you’re going to feel lonely and then if there is a friend of yours beside you, someone to lean on, so you got to know that there’s life after this …’ (FG 4, P69, YMLWH, age 18)

### Learning to live with HIV

As the young people began to experience acceptance and support from significant others and/or friends, they began to feel motivated to learn about ART. The information that HIV was a treatable condition and that adherence would minimise sickness and prevent early death gave young people a sense of control and power over their condition, which in turn enabled them to integrate living with HIV into their daily routines. For this young woman, this knowledge became a turning point in her life:

‘I just accept it, then you take it as a living thing that you are living within you. … everything that appears in your body that just take easy and just live your normal life … it’s not a death sentence just like everyone just said now … it’s not the end of the world. … It’s just a step back for a come-back …’ (FG 4, P54, YWLWH, age 23)

Knowing that HIV was a manageable chronic condition allowed this young woman to cope:

‘… If you normally take your pills right and … you take your pills like your blood pressure … you’re fine. It’s not a monster that controls you, and you take that away, you take the power of it away.’ (FG 4, P59, YWLWH, age 21)

#### Learning about ART in the family

As family members accepted a young person’s diagnosis, they in turn sought treatment information from the clinics to understand how to support their loved one:

‘My mother accepted my status, and while I was sick, she went to the clinic to ask about the pills, so she could support me. From then she made sure I took the pills every day, my mother saved my life.’ (FG 4, P28, YWLWH, age 21)

For this participant, however, lack of understanding had negative consequences:

‘When I was first told I have AIDS I was sick at the hospital and they gave me the pills. I got better and told my father who supported me. At home I took the medicine every day and my father always asked, have you eaten the pills? Then when I was very well, better and looked fat again. He said, why do you still take the pills? You are better now; you don’t need to keep taking the pills. So, I stopped.’ (Clinic 1 observation, P83, YWLWH, age 17)

#### Learning to live with HIV as a community

Young people who tested negative for HIV seldom had further contact with the health services until they returned for another HIV test. Support groups for PLWH were viewed as a form of segregation as participants felt that everyone should be able to attend so that they too could learn about HIV and ART. Knowledge enabled them to support their peers when diagnosed as HIV-positive and challenge discrimination:

‘I was going to say it’s better if you bring all the people together, not the positive alone, because when they are alone, they are like separated, it’s like they’re not a part of the community any more, you know.’ (P66, M, FG4, age 18)

‘I think it [*would*] take out the stigma.’ (P68, M, FG4, age 17)

‘Ja, to give them knowledge you know, because they behave in certain ways because they don’t have the knowledge of HIV. Some people commit suicide.’ (P72, M, FG4, age 19)

## Discussion

**FIGURE 1:**
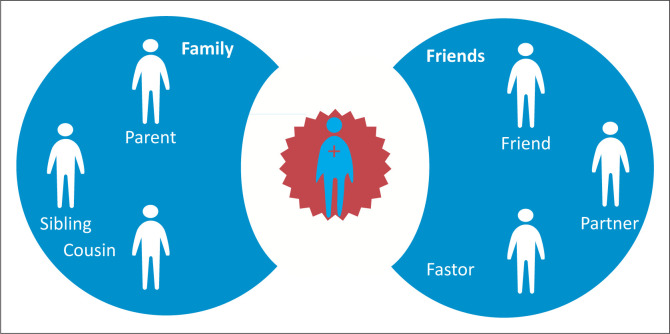
Response to diagnosis.

An understanding of how young people become motivated to learn about ART and adhere to the regime has emerged from the findings. The main determinant of adherence motivation after being diagnosed with HIV was reconnecting to at least one of participants’ significant others. The diagnosis threatens the young person’s perceptions about self-worth, identity and sense of belonging. As the young person begins to feel accepted by his or her ‘belonging group’, hope is renewed and motivation to learn about and adhere to the ART regime increases, as illustrated in Figure 1.

The three elements of trauma upon diagnosis concur with the findings of other studies (Anderson et al. 2010; Martin & Kagee 2011; Martinez et al. 2012). Catastrophic thinking is a common response to trauma that can generate both situational and over-generalised fear, which may be imagined or based on real-life experiences and is especially relevant for persons receiving a diagnosis of HIV (Baumgartner & David 2009). The experience of many young South Africans would have included the suffering and death of a close relative from HIV (Petersen et al. 2010) and exposure to historical narratives of HIV- and AIDS-related deaths. This study found that when young people are informed of the HIV-positive diagnosis, traumatic memories of loss may be rekindled, which prevent any engagement with health information about HIV, treatment protocols and linkage to care plans.

Healthcare workers attempt to dispel the initial fear and despair felt by the newly diagnosed young persons, through reassurance and positive reinforcement about the benefits of treatment and adherence. This approach is typical of the HBM that centralises the benefits of a health intervention or treatment as the key determinant to becoming motivated towards a proposed behaviour (Champion & Skinner 2008). The findings showed, however, that understanding of treatment, although important for achieving adherence, is not the main motivational factor for young people. The use of health behaviour model frameworks may therefore limit a holistic understanding of localised meanings of what motivates people to adhere to treatment.

Concerns about partner infidelity (Ruark et al. 2019) and intimate partner violence with respect to STI and HIV disclosure (Durevall & Lindskog 2015) have been reported. Stigma in relation to HIV has been widely studied, and despite general awareness of HIV and its treatment stigma remains a concern for many PLWH (Gilbert & Walker 2010; Nglazi et al. 2012). Young people described stigma as a fear of discrimination and feelings of internalised shame and blame, referred to by Gilbert and Walker (2010) as ‘felt’ stigma.

These findings can be understood in the context of the developmental journey of adolescents and young people, as they seek to develop an autonomous self-identity as described by Erikson (1980). According to Erikson, individuation is not an isolated process; the emerging autonomous self requires affirmation and acceptance from both the young person’s primary caregivers and close friends, enabling them to validate their emerging self-identity as an ‘I’ (authentic me) in the context of their social world. From a symbolic interactionist perspective, self-identity is a self-perception that is adopted through the act of interaction with others (Charon 2010). It is from these places of belonging that self-identities are formed and where their meaning is lived relationally, both in the present and in the imagined futures that are the source of human motivation (Solomon & Siegel 2003).

Social rejection that disconnects young persons from their sense of belonging and decreased meaningfulness may lead to a state of severe deprivation (Twenge et al. 2007), which in turn affects adherence behaviour. Lall et al. (2015) refer to the dual stigma of adolescents for relating to their HIV status as well as belonging to a marginalised population and argue that access to psychosocial support, experience of stigma and access to social and behavioural support are amongst the important determinants of adherence behaviours.

The findings show that an HIV diagnosis threatened the young people’s sense of belonging, health status and the potential for meaningful relationships. Post-diagnosis their main concern was to reconnect with significant other(s) to receive their acceptance and support. A young person’s process of acceptance of an HIV-positive diagnosis is inextricably linked to a sense of being accepted and belonging. An essential part of the process of meaning-making within a specific situation occurs through the process of self-interaction, which then directs a person’s behaviour (Scott 2015). Emotional responses are also an intrinsic element of self (Scott 2015) and defined how the participants responded to their newly discovered HIV status. Interpretations of emotions are based on perceived or imagined emotional responses of significant others (Charon 2010), whose perspective is most valued because of their significance to the individual. This then becomes the principal determinant of how a person defines and behaviourally responds to a situation (Charon 2010).

Our study has shown that young people’s perceptions of a highly stigmatised disease and the responses from their belonging group determine their behavioural responses to their diagnosis. The act of disclosing an HIV diagnosis to a family member is associated with the risk of rejection. For young people, to be separated from their core belonging group was to be cast out, to become someone without identity, purpose and hope (see Figure 1).

Figure 1 shows how a young person feels isolated and disconnected from their belonging and associated persons immediately after receiving an HIV-positive diagnosis.

**FIGURE 2:**
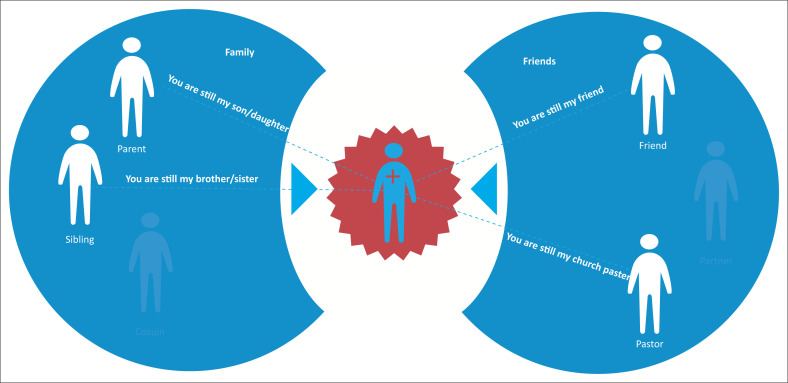
Post-diagnosis messages.

Soon after diagnosis, young people needed to reconnect with trusted members of their belonging group, who through acceptance and support affirm them as someone who has not fundamentally changed; they were still a son, daughter, brother, cousin and friend (see Figure 2).

Figure 2 shows the messages that young people want to hear from belonging group members post-diagnosis.

Families and significant others need to recognise the importance of a sense of belonging for young people’s overall well-being. Young people need support to cope with the trauma response of an HIV diagnosis, to find personal acceptance and to feel accepted by trusted members of their belonging group.

Health communication strategies, therefore, need to move beyond disseminating messages that focus only on the bio-medical aspects of HIV, such as prevention and treatment, which are typically individualistic and cognitive. Strategies should include proactive engagement with wider communities to encourage acceptance and support and to facilitate better communication between young people and their belonging groups.

**FIGURE 3:**
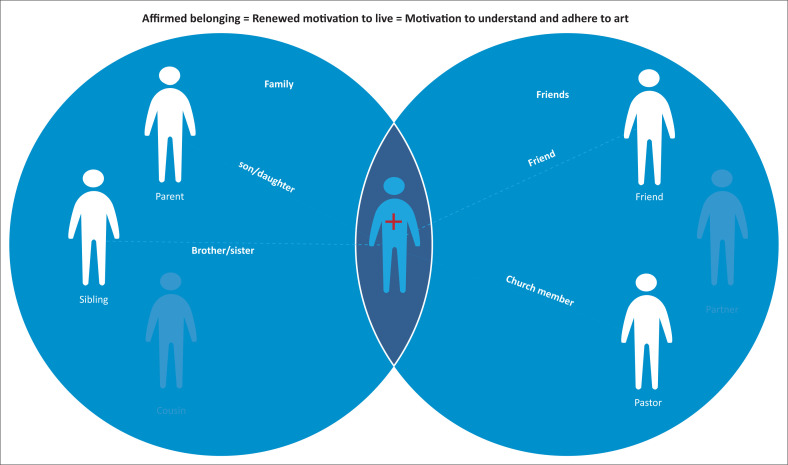
Affirmation.

As young people experience acceptance, affirmation and support, they begin to reconnect with their belonging group and find renewed hope for living. This facilitates their motivation to learn about ART to ensure that they stay healthy and well, enabling them to enjoy their lives and the activities of daily living (see Figure 3). During this process of reconnection, family member(s) can also become motivated to learn about ART. For the participants, it was important that their family members also learn about treatment to better support adherence. Treatment knowledge is relevant not only for the PLWH but also for their belonging group. Self-efficacy is more likely to occur when a supportive individual or social group encourages a proposed health behaviour (Qiao, Li & Stanton 2014).

Figure 3 shows how affirming message helps reconnect a young person to their sense of belonging that is the central driver to their motivation to learn about treatment and adhere to it.

Treatment knowledge enabled family members to offer solidarity with their loved one, which Molm, Collett and Shaefer (2007:207) describe as ‘the integrative bonds that develop between persons and social units to which they belong’. Solidarity was evident in the group approach of young people who visited the clinics in groups to be tested. They wanted to learn about treatment irrespective of their test result, to be able to support a family member or trusted friend who became infected and to enhance coping should they themselves ever test positive. Increased knowledge of HIV and ART is associated with a decrease in stigma and reduction of the fear of HIV and of those who lived with HIV (Mall et al. 2013). The process of reducing stigma could begin by providing opportunities for young people to learn and talk about HIV and ART treatment in groups where HIV status is not prioritised nor made a pre-condition of attendance. Adherence communication strategies should explore ways of harnessing the enthusiasm and desire of young people who want to learn about treatment, irrespective of status. This could contribute to the reduction of stigma amongst young people and the wider community.

## Recommendations

A communication strategy that motivates adherence must facilitate and support young people to reconnect to trusted members of their belonging groups. It should also support belonging group members to accept, affirm and learn how to support their loved ones on the adherence journey. Communication resources should be made available for belonging group members. This could facilitate the process of acceptance of a family member’s HIV status and enable them to provide support.

An adherence communication strategy should also engage the wider community, aiming to increase treatment knowledge and encourage acceptance and support as a community response to HIV discrimination and stigma. The importance of *belonging* in the context of young people’s well-being should be an integral component of health promotion strategies. Antiretroviral therapy messages and linkage to care instructions should be carefully communicated before an HIV test, ensuring that key information is understood, as young people find it difficult to meaningfully engage because of post-diagnosis trauma.

These findings are unlikely to be unique to this setting or to HIV and have broader implications for the future development of social behavioural communication strategies that are focussed on increasing young people’s motivation to learn about and adhere to ART.

## Limitations

This study was limited to predominantly isiXhosa-speaking young people aged 16–24 years in a specific geographic area, who were in contact with the health services. In researching sensitive topics, there is potential for response bias, and we cannot claim that all contributions were truthful or represented participants’ genuine feelings or experiences.

## Conclusion

The study offers a new theoretical understanding that places belonging as the main determinant of motivation to learn about ART and adhere to the treatment programme. Adherence is a social construction that is dependent upon a series of interpersonal communications between young people and members of their belonging group. Communication about HIV and adherence to young people should be based on the understanding of the young person’s diagnosis journey.

For young people, the impact of receiving an HIV-positive diagnosis is overwhelming, traumatising, engendering fear of illness and an early death, as well as rejection by family and trusted friends. To be separated from their core belonging group is to be rejected, to become someone without identity, purpose and hope. Soon after diagnosis, young people needed to reconnect and be accepted by trusted members of their belonging group. They are then affirmed as still a valued and loved family member and friend. When a young person receives acceptance and support, he or she becomes reconnected to the sense of belonging that in turn enables the young person to find meaning and motivation for living in the present and see a future. It is *this* motivation to *live* that becomes the central driver for becoming motivated to learn about and adhere to treatment.
